# Epigallocatechin-3-gallate confers protection against corticosterone-induced neuron injuries via restoring extracellular signal-regulated kinase 1/2 and phosphatidylinositol-3 kinase/protein kinase B signaling pathways

**DOI:** 10.1371/journal.pone.0192083

**Published:** 2018-01-26

**Authors:** Xiaoling Zhao, Renjia Li, Hui Jin, Haimin Jin, Yonghui Wang, Wanqi Zhang, Haichao Wang, Weiqiang Chen

**Affiliations:** 1 Tianjin Institute of Health and Environmental Medicine, Tianjin, China; 2 Tianjin Medical University, Tianjin, China; 3 Yan’an Hospital of Kunming City, Kunming, Yunnan province, China; 4 The Feinstein Institute for Medical Research, Manhasset, NY, United States of America; 5 Affiliated Hospital of Taishan Medical University, Taian, Shandong province, China; 6 Department of Emergency Medicine, North Shore University Hospital, Manhasset, NY, United States of America; University of PECS Medical School, HUNGARY

## Abstract

Extensive studies suggested epigallocatechin-3-gallate (EGCG) has significant neuroprotection against multiple central neural injuries, but the underlying mechanisms still remain poorly elucidated. Here we provide evidence to support the possible involvement of extracellular signal-regulated kinase 1/2 (ERK1/2) and phosphatidylinositol-3 kinase/ protein kinase B (PI3K/AKT) pathways in EGCG-mediated protection against corticosterone-induced neuron injuries. As an essential stress hormone, corticosterone could induce obvious neurotoxicity in primary hippocampal neurons. Pre-treatment with EGCG ameliorated the corticosterone-induced neuronal injuries; however, it was blocked by pharmacological inhibitors for ERK1/2 (U0126) and PI3K/AKT (LY294002). Furthermore, the results confirmed that EGCG restored the corticosterone-induced decrease of ERK1/2 and PI3K/AKT phosphorylation, and attenuated the corticosterone-induced reduction of peroxisome proliferators-activated receptor-γ coactivator-1α (PGC-1α) expression and ATP production. Taken together, these findings indicated that EGCG has significant neuroprotection against corticosterone-induced neuron injuries partly via restoring the ERK1/2 and PI3K/AKT signaling pathways as well as the PGC-1α-mediated ATP production.

## Introduction

As the most abundant polyphenol in green tea, epigallocatechin-3-gallate (EGCG) has been shown to exhibit beneficial effects against diverse central neural injuries, such as neurodegenerative diseases [1–3], stress-induced neural injuries [4, 5], cerebral ischemic injuries [6, 7], neuro-inflammation [8, 9], and neural injuries induced by toxic reagents [10]. EGCG is the main active component of green tea polyphenols (GTPs), accounting for 30–50% of the total catechins [11]. Due to the abundance of phenolic hydroxyl groups in its chemical structure, EGCG is generally considered as one of the most important naturally occurring anti-oxidant [12]. Besides its powerful anti-oxidative properties, EGCG also plays an important role in modulating metal-chelation [13], anti-apoptosis [14], mitochondrial-preservation [15], and cellular signal transductions [1, 14, 16]. At the present, there remains no effective medical treatment for neurodegenerative diseases and other central neural injuries [17]. Most of the commercial therapeutic agents are predominantly symptom-oriented and accompanied with lots of side effects [18]. Therefore it’s urgent and necessary to explore and develop some therapeutic agents with lower side effects and wider spectrum of targets to not only treat the symptoms but also potentially modulate the pathology of such diseases and dysfunctions. As EGCG possesses multifunctional bioactivities, more and more attentions are paid to extensively investigate EGCG as a good candidate for a potent disease-modifying agent with neuro-rescue and neuro-protective properties.

Despite recent progress, more efforts are still needed to elucidate the molecular mechanisms underlying EGCG-mediated neuroprotection. Studies suggested that EGCG interacts directly with some neurotransmitter receptors, downstream protein kinases and stress-sensitive signaling cascades such as protein kinase C (PKC), protein kinase B (PKB/AKT) and Mitogen-activated protein kinase (MAPK) signaling pathways, which further dictates the neuronal cellular response to stress, thereby affecting cell proliferation, apoptosis, synthesis of inflammatory mediators and neurite growth [19, 20]. Our latest study indicated that EGCG protects against stress-induced central neural injuries by enhancing extracellular signal-regulated kinase 1/2 (ERK1/2) and PKCα signaling [4]. To further determine EGCG’s prominent regulating properties on stress-induced cellular signaling alternations and identify the potential molecular targets of EGCG-mediated neuroprotection, we conducted the present study. As an essential stress hormone, corticosterone (CORT) exposure could cause significant cytotoxicity including DNA damage, differential protein activation and cell apoptosis [21, 22]. The molecular mechanisms underlying the CORT-induced neuronal injuries were partly dependent on the inhibition of ERK1/2 and phosphatidylinositol-3 kinase/ protein kinase B (PI3K/AKT) pathways [23–25]. Therefore, in this study we employed corticosterone to induce neuronal stress in primary rat hippocampal neurons, and the modulating effects of EGCG on two stress-susceptible signaling pathways, namely ERK1/2 and PI3K/AKT pathways were examined in vitro to elucidate EGCG’s neuroprotective mechanisms.

## Materials and methods

### Chemical and reagents

EGCG (purity ≥ 99% by high-performance liquid chromatography) was provided by Hangzhou Hetian Biotech Co., Ltd (Hangzhou, Zhejiang Province, China). Neurobasal medium, Dulbecco’s modified eagle’s medium (DMEM), B27 supplement, and fetal bovine serum (FBS) were purchased from Invitrogen (Carlsbad, CA, USA). Corticosterone (CORT), trypsin, poly-L-lysine, hoechst33342, and 3-(4, 5-dimethyl thiazol-2-yl)-2, 5-diphenyltetrazolium bromide (MTT) were purchased from Sigma (St. Louis, MO, USA). LY294002 (an inhibitor of PI3K/AKT), U0126 (an inhibitor of MEK/ERK1/2), L-glutamate, and antibodies against ERK1/2, phospho-ERK1/2 (pERK1/2), AKT, phospho-AKT (Ser473), PGC-1α and β-actin were purchased from Cell Signaling Technology (Danvers, MA, USA). ATP assay kit (ab83355) was purchased from Abcam (Cambridge, MA, USA). Ultrapure RNA kit was purchased from Beijing Kangwei Century Company (Beijing, China). All-in-one™ First Strand cDNA synthesis kit was provided by Guangzhou GeneCopoeia Co., Ltd, (Guangzhou, China).Other chemicals and reagents were of the highest analytic grade and were purchased from Beijing Chemical Reagent Company (Beijing, China).

## Cell culture

The newborn (postnatal day 1) Wistar rats were obtained from the Experimental Animal Center of the Academy of Military Medical Sciences. All experimental procedures were taken in accordance with Tianjin Institute of Health and Environmental Medicine experimental standards as well as international guidelines on the ethical treatment of laboratory animals. Primary hippocampal neuronal cultures were prepared as described previously [26] with some modifications. Briefly, after treatment with 0.125% trypsin for 15 min at 37°C in Ca^2+^ and Mg^2+^ free Hank’s balanced salt solution, the hippocampi were washed in DMEM with 10% FBS in order to stop trypsin activity. Then the cells were re-suspended in DMEM supplemented with 10% FBS and plated onto poly-L-lysine-coated plates for 24 h at 37°C in a humidified atmosphere of 95% air and 5% CO_2._ Following cellular attachment, the culture medium was replaced with neuronal culture medium, namely serum-free Neurobasal medium with 2% B27 supplement, 0.5 mM glutamine, 100 U/mL penicillin and 100 U/mL streptomycin, followed by re-incubation for 7–8 days, the time required for maturation of hippocampal neurons, with half of the medium being changed every 3 days. Then, the cells were characterized by immunocytochemistry for neurofilament protein and fibrillary acidic protein, revealing that the cell cultures contained about 98% neurons.

### Cell treatment

EGCG at different concentrations (0.1, 1, 5, 10 μmol/L) was added to primary hippocampal neuron cultures respectively 1 h before exposure to 10 μmol/L CORT and allow to co-incubation for an additional 24 h at 37°C in a humidified incubator of 5% CO_2,_ 95% air environment, followed by various assessments at desired time. The hippocampal neurons undergoing neither EGCG pretreatment nor CORT stimulation served as control.

To investigate the involvement of PI3K/AKT and ERK1/2 signaling in EGCG-mediated neuroprotective effects, specific inhibitors for PI3K/AKT (LY294002, 10 μmol/L) or MEK/ERK1/2 (U0126, 10 μmol/L) were added 30 min before CORT-treatment in the absence or presence of EGCG (0.1 μmol/L), and further incubated for 24 h.

### Cell viability measurement

Neuronal cell viability was determined by MTT assay based on the cleavage of the yellow tetrazolium salt MTT to purple formazan by mitochondrial enzymes in metabolically active cells. In brief, hippocampal neurons were cultured in 96-well plate at a density of 5×10^5^ cells per well. After stimulation with CORT, the cells were further incubated with the neuronal culture medium for a certain time period, and then 10 μl MTT solution were added to each well, and incubated at 37°C for 4 h. Afterwards, 100 μl of 20% sodium dodecyl sulfide (SDS) was added to dissolve the resulting formazan. The absorbance (OD) values were measured by spectrophotometry at 570 nm with an EIX-800 Micro-ELISA reader (Bio-Tek Inc., Winooski, VT). The cell viability data were expressed as a percentage of control value.

### Hoechst staining

The hippocampal neurons were fixed in 4.0% paraformaldehyde for 20 min and stained with 5μg/ml Hoechst 33342 dye at 37°C for 10min, followed by observation under a DMR fluorescence microscope (Leica Microsystems, Wentzler, Germany) with fluorescence excitation at 340 nm and emission at 510 nm. In order to quantify the apoptotic process, cells with fragmented or condensed DNA and normal DNA were respectively counted to calculate the ratio of apoptotic cells to total cells.

### Western blot analysis

The cultured hippocampal neurons were subjected to western blot analysis for determining some proteins’ levels as per routine procedures. Briefly, cells were harvested and lysed in a lysis buffer. Protein concentrations were quantified using the BCA assay kit, followed by electrophoresis separation on 10% SDS-PAGE. After transferring to PVDF membranes, blocking with 20% bovine serum albumin(BSA) in Tris-buffered saline containing 0.05% Tween-20(TBST) for 2 h at room temperature, the protein membranes were allowed to react with the respective primary monoclonal antibody overnight at 4°C. The primary antibodies and concentrations were ERK1/2 (1:1000); pERK1/2 (1:1000); AKT (1:1000); p-AKT (1:1000); PGC-1α (1:1500) and β-Actin (1:1000), respectively. β-actin was used as an internal loading control. Membranes were washed with TBST 5 times, and then probed with horseradish peroxidase (HRP)-conjugated secondary antibody (1:5000 dilutions in TBST) for 1 h at room temperature with gentle shaking. Membranes were washed with TBST, and the immuno-reactivity was detected with the Enhanced Chemiluminiscence (ECL) Western Blot Detection system (WEST-ZOL®plus) and visualized with “ChemiDoc XRS” digital imaging system. Then protein expression was quantitated densitometrically with ‘MultiAnalist’ software from Bio-Rad laboratories Inc.

### Detection of ATP contents in culture neurons

The neuron cells of hippocampus were homogenized and ultra-sonicated for 1.5 min, and centrifuged at 15,000 g at 4°C for 10 min. The supernatant was assayed for ATP productions by using an ATP Assay Kit according to the manufacturer’s instructions.

### Real-time RT-PCR

Total RNA was isolated from the primary cultured hippocampal neurons using the Ultrapure RNA Kit according to the manufacturer’s instructions, and its purity was confirmed by the A260/A280 ratio. Then the mRNA was reversely transcribed into the first-strand cDNA using All-in-one™ First Strand cDNA synthesis Kit. Following reverse transcription, All-in-one qPCR Primer (2 μM) and primers for glyceraldehyde 3-phosphate dehydrogenase gene (Gapdh; Qiagen, QT01658692) were used to quantify the mRNA expression levels of respective genes using an ABI 7300HT Real-time PCR system (Applied Bio-systems, Foster City, CA, USA). Amplification was performed using the RT^2^ SYBR Green ROX qPCR Mastermix under the following conditions: 95°C 10 m; followed by 40 cycles of 95°C for 10 s, 60°C for 20 s and 72°Cfor 15 s. Immediately following the amplification step, a single cycle of the dissociation (melting) curve program was run at 95°C for 15 s, at 60°C for 20 s, then at 95°C for 15 s, and last at 60°C for 15 s. This cycle was followed by a melting curve analysis; baseline and cycle threshold values (Ct values) were automatically determined using the ABI 7300HT software. The relative mRNA expressions were calculated using the following formula: ΔΔC expression = 2^-ΔΔCt^, where ΔΔCt = ΔCt (modulated group)– ΔCt (control group), ΔCt = Ct (target gene)–Ct (GAPDH) and Ct = cycle at which the threshold was reached. The relative abundance of mRNA expression in normal control group was set as an arbitrary unit of 1, and the gene expression in modulated groups was presented as folds of controls after normalization to GAPDH.

### Statistical analysis

The statistical analysis was performed using SPSS 10.0 software, and the results were expressed as mean ± SD (standard deviation). Experimental data were checked for Gaussian distribution. Two-tailed unpaired Student t tests were applied for comparison of two normally distributed groups; comparisons between more than two normally distributed groups were made by one-way ANOVA followed by pairwise multiple comparison (Student-Newman-Keuls method, q-test). Difference was considered statistically significant at *P*<0.05.

## Results

### Establishment of neuron stress model and EGCG’s protection in vitro

**Fig 1:**
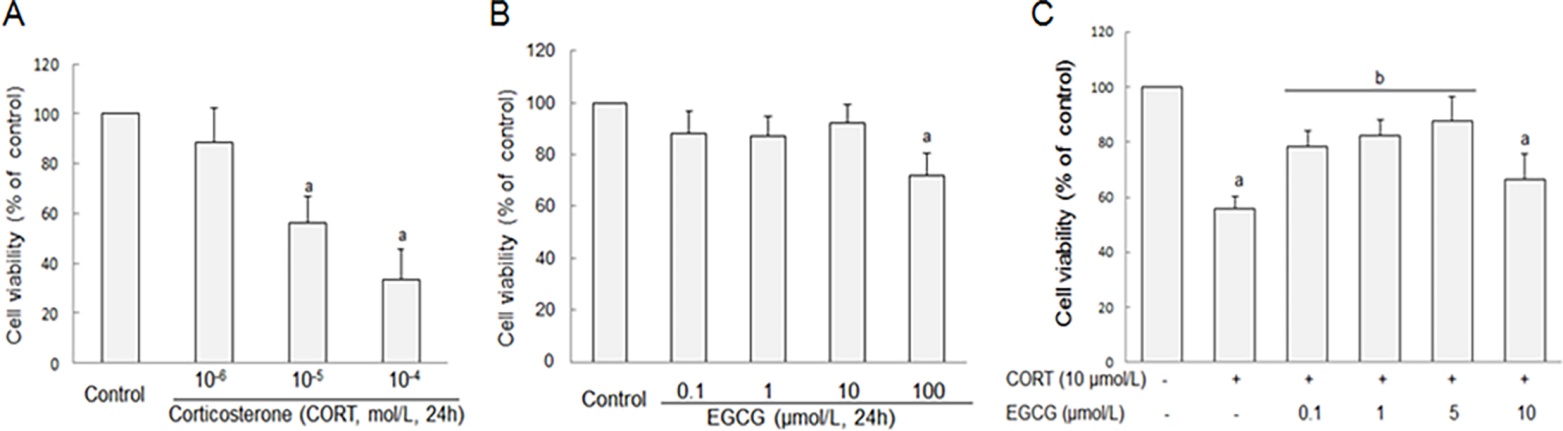
The effect of CORT and EGCG on cell viability of primary hippocampal neuron cultures. Primary cultured hippocampal neurons were treated with various concentrations of CORT for 24 h and cell viability was assessed by MTT (A). Hippocampal neurons were exposed to different concentrations of EGCG for 24 h and cell viability was assessed by MTT (B). Hippocampal neurons were pretreated with different concentrations of EGCG for 30 min before subsequent stimulation with CORT for 24 h (C). ^a^ *P*<0.05 *vs* Control group; *P*<0.05 *vs* CORT group.

After treatment with CORT for 24 h, the hippocampal neurons demonstrated significant decrease of cell viability in 10 μmol/L and 100 μmol/L CORT treated groups (Fig 1A). At the concentration of 10 μmol/L, CORT treatment resulted in a decrease of relative cell viability to 56.3±10.8% in cultured hippocampal neurons. Thus, this concentration of CORT was used to induce neuron injuries in the subsequent experiments. When normal neurons were treated with EGCG for 24 h at different concentrations, there were no significant changes in cell viability if EGCG was given at concentrations < 100 μmol/L, indicating that EGCG itself exerts no toxicity on hippocampal neurons when given at reasonable doses (Fig 1B). Furthermore, EGCG evidently attenuated the CORT-induced decrease of cell viability in a dose-dependent manner between 0.1 and 5 μmol/L concentration while it remained ineffective at 10 μmol/L level (Fig 1C). As predicted, at extremely high concentration (100 μmol/L), EGCG itself caused a decrease of cell viability (Fig 1B).

**Fig 2:**
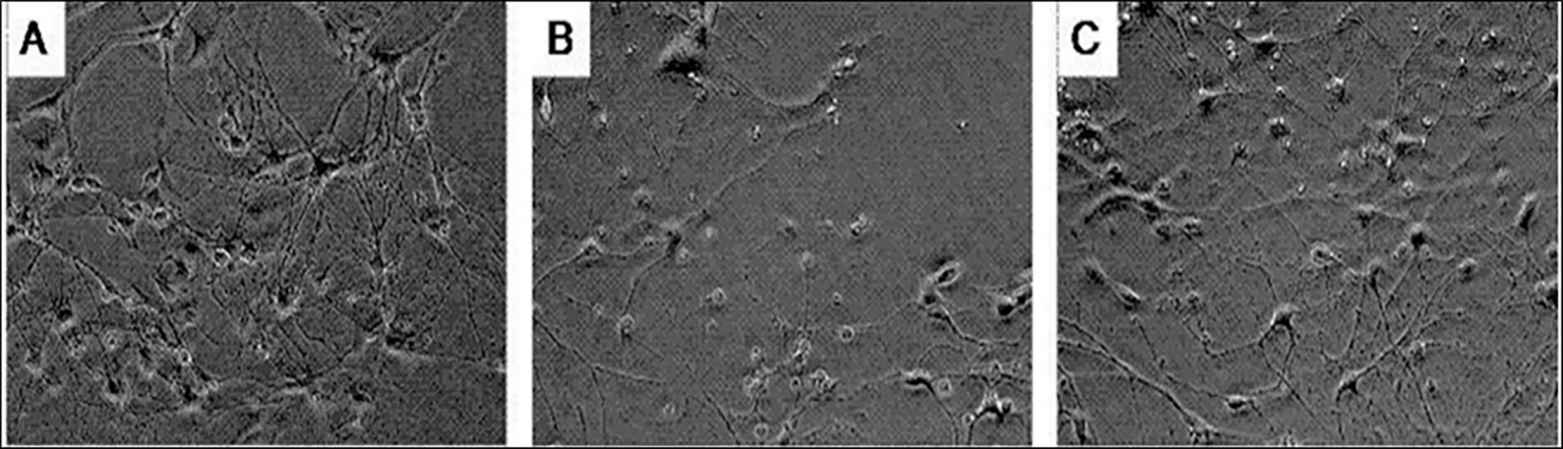
The changes of cell morphology of primary cultured hippocampal neurons. Primary hippocampal neuron cultures were treated with CORT (10 μmol/L) for 24 h in the absence or presence of EGCG pretreatment (1μmol/L, at 30 min prior to CORT addition). A) Normal neurons, ×200; B) CORT treated neurons, ×200; C) EGCG co-incubation with CORT-treated neurons, ×200.

In addition, the CORT-treated neurons displayed a significant morphological change, such as the disappearance of neurite, emergence of vacuoles around the cell body and decrease of cellular refraction (Fig 2B), while co-incubation with EGCG markedly attenuated these CORT-induced morphological changes (Fig 2C).

### The effects of ERK1/2 and PI3K/AKT inhibition on EGCG-mediated neuroprotection

**Fig 3:**
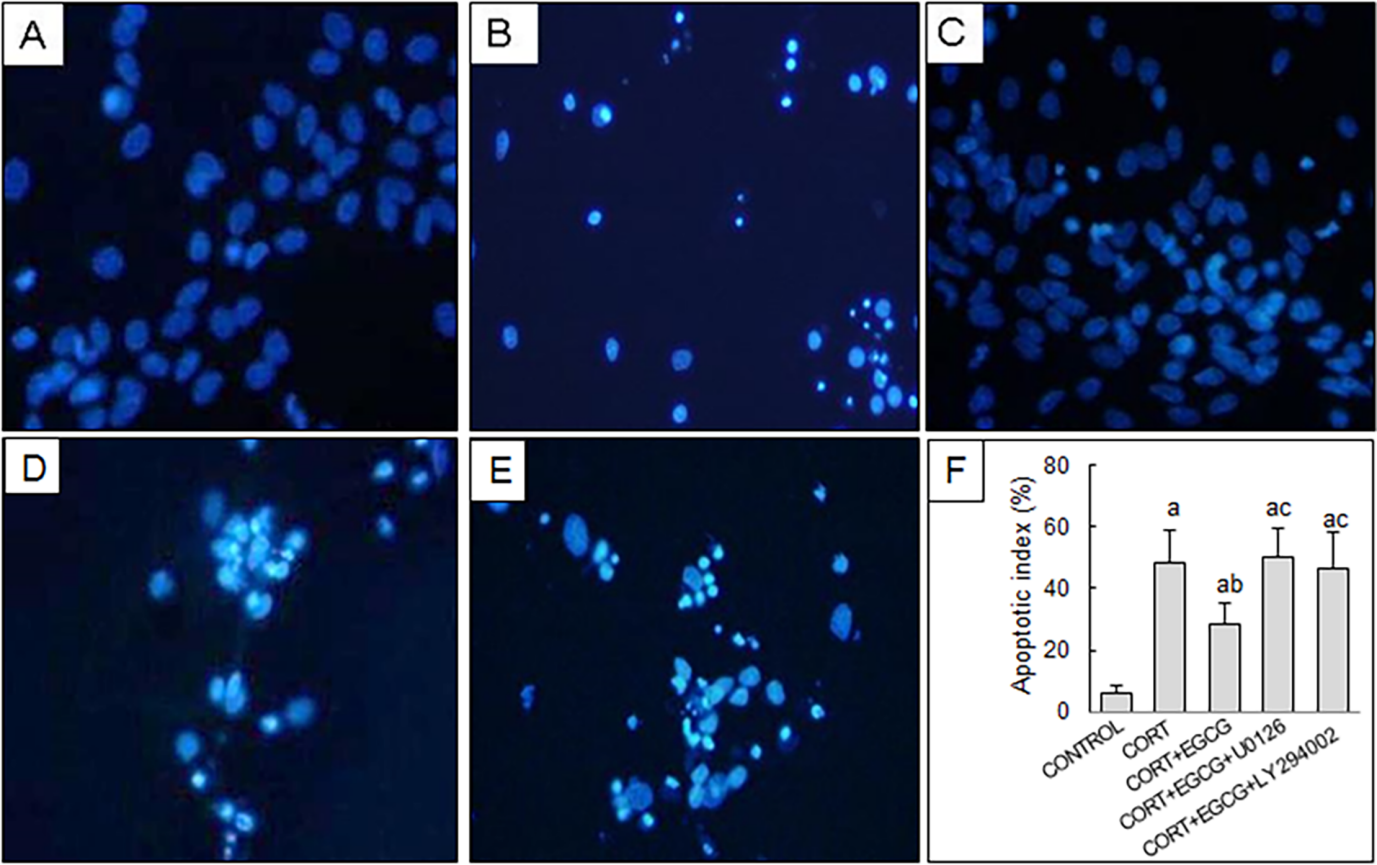
The effect of EGCG on CORT-induced morphological changes of hippocampal neurons by Hoechst 33342 staining. Primary hippocampal neuron cultures were treated with CORT for 24 h in the absence or presence of EGCG pre-treatment (1 μmol/L, 2 h prior to CORT stimulation), ERK1/2 inhibitor (U0126, 10 μmol/L) or PI3K/AKT inhibitor (LY294002, 10 μmol/L) pre-treatment (30 min before CORT exposure). A) Normal control neurons; B) CORT treatment; C) EGCG and CORT co-treatment; D) EGCG+CORT+U0126 treatment; E) EGCG+CORT+ LY294002 treatment; F) The apoptotic index of hippocampal neurons. ^a^ *P*<0.05 *vs* Control group: ^b^ *P*<0.05 *vs* CORT group; ^c^ *P*<0.05 *vs* CORT+ EGCG group.

To elucidate the molecular mechanisms underlying EGCG-mediated neuroprotection, we determined the involvement of several signaling molecules using specific pharmacological inhibitors. The co-incubation with inhibitors specific for ERK1/2 (U0126) or PI3K/AKT (LY294002) signaling pathways markedly abolished EGCG-mediated neuroprotective effects, as the cell viability remarkably declined compared with that of normal control group or EGCG/CORT-treated group (Table 1). By itself, U0126 or LY294002 did not affect cell viability in the primary hippocampal neurons in the absence of CORT or EGCG. The results of Hoechst 33342 staining of hippocampal neurons showed that EGCG improved the neuron injuries induced by CORT, such as nuclear shrinkage, pyknosis fragmentation and appearance of apoptotic bodies (Fig 3C). However, EGCG’s protective effects were impaired by co-incubation with U0126 or LY294002 (Fig 3D and 3E), as judged by the number of apoptotic bodies or calculated apoptotic index (Fig 3F).

**Table 1:** The effects of U0126 and LY294002 on EGCG-mediated neuroprotection against corticosterone-induced cell death (mean ± standard deviation).

Group	Cell viability (%)
Normal control	100
CORT (10 μmol/L)	69.39 ± 7.63^a^
U0126 alone (10 μmol/L)	98.54 ± 7.27
LY294002 alone (10 μmol/L)	97.79 ± 8.76
CORT(10 μmol/L) +EGCG(1 μmol/L)	90.22 ± 9.41^b^
CORT(10 μmol/L) +EGCG(1 μmol/L)+ U0126 (10 μmol/L)	62.54 ± 10.22^a^^c^
CORT(10 μmol/L) +EGCG(1 μmol/L)+ LY294002 (10 μmol/L)	66.90 ± 3.78^a^^c^

Primary hippocampal neuron cultures were treated with CORT for 24 h in the absence or presence of EGCG (1 μmol/L) pre-treatment (2 h prior to CORT stimulation), ERK1/2 inhibitor (U0126, 10 μmol/L) or PI3K/AKT inhibitor (LY294002, 10 μmol/L) pre-treatment (30 min before CORT exposure).

^a^ *P*<0.05 *vs* Normal control group

^b^ *P*<0.05 *vs* CORT group

^c^ *P*<0.05 *vs* CORT +EGCG group

### CORT induces dynamic activation of ERK1/2 signaling pathway in primary hippocampal neuron cultures

**Fig 4:**
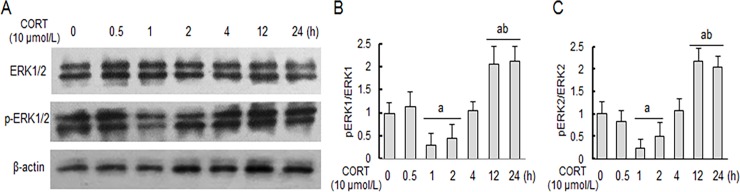
Corticosterone induces dynamic changes of ERK1/2 phosphorylation in primary hippocampal neuron cultures. Primary hippocampal neuron cultures were stimulated with CORT for indicated time period, and the cellular levels of total and phosphorylated ERK1/2 were determined by western blot analysis with reference to a house-keeping protein (β-actin) as loading control. The specific signals were visualized with “ChemiDoc XRS” digital imaging system, and a representative western blot was shown (A). The relative protein levels were expressed as the relative band density of the corresponding protein (B, C). ^a^ *P*<0.05 *vs* normal control group: ^b^ *P*<0.05 *vs* CORT treatment 2 h group.

**Fig 5:**
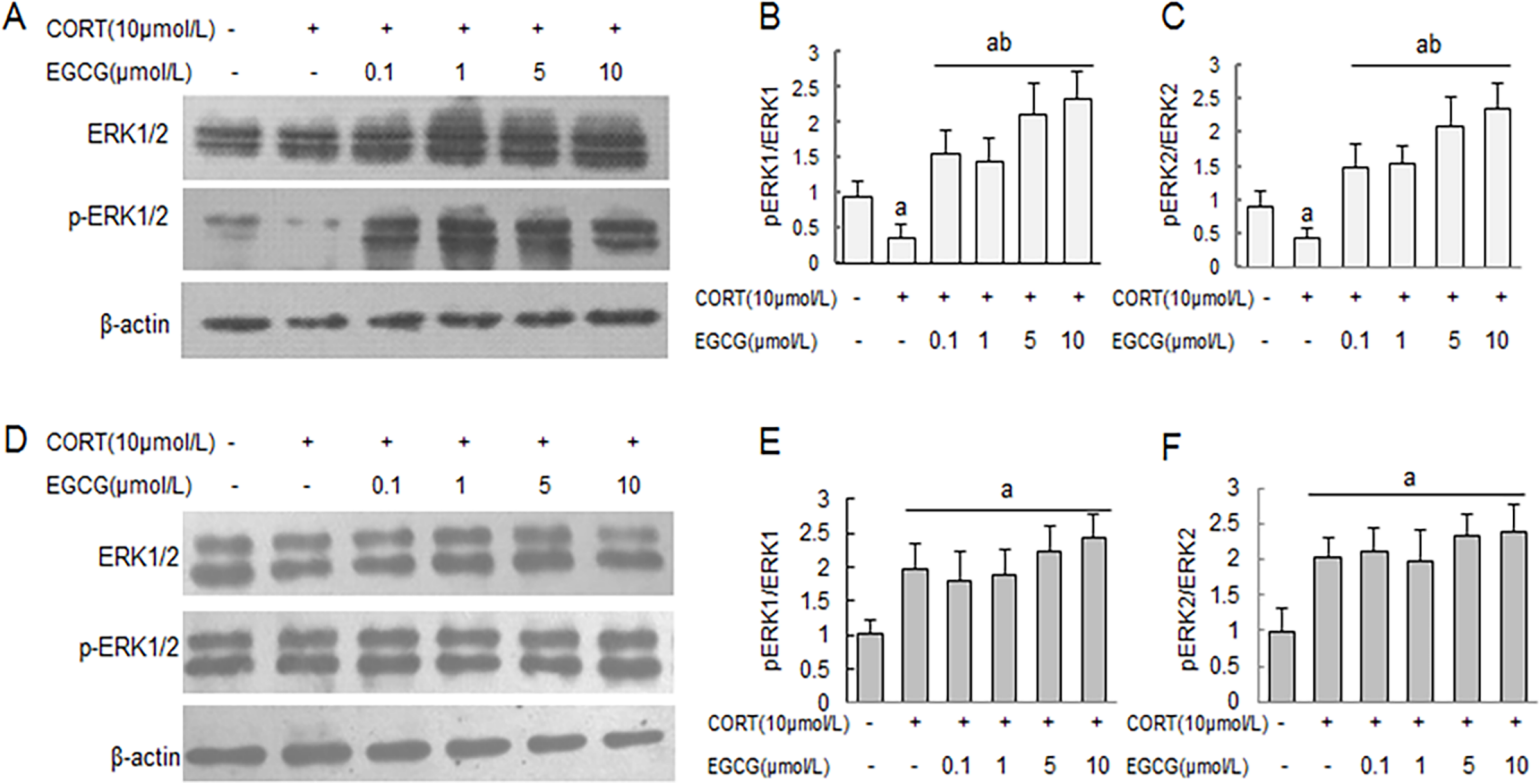
The effect of EGCG on CORT-induced alteration of ERK1/2 phosphorylation in primary hippocampal neuron cultures. Primary hippocampal neuron cultures were pre-treated with EGCG at indicated concentrations for 2 h before stimulating with CORT for 1 h (A) and 24 h (D), respectively. The cellular levels of total and phosphorylated ERK1/2 were determined by western blot analysis with reference to a house-keeping protein (β-actin) as loading control. The relative protein levels were expressed as the relative band density of the corresponding protein (B, C, E and F). ^a^ *P*<0.05 *vs* normal control group: ^b^ *P*<0.05 *vs* CORT treatment group.

Following stimulation with CORT (10 μmol/L) for different time period, the levels of phosphorylated ERK1/2 in primary hippocampal neuron cultures were altered in a time-dependent fashion, first reduced between 1–2 h, but subsequently elevated between 12–24 h (Fig 4). The co-incubation with EGCG obviously prevented the CORT-induced early inhibition of ERK1/2 phosphorylation in a dose-dependent manner (Fig 5A, 5B and 5C). Furthermore, the ERK1/2 phosphorylation levels in EGCG treatment neurons were enhanced significantly compared with that of normal control neurons (Fig 5A, 5B and 5C). In a sharp contrast, EGCG did not affect the CORT-induced elevation of ERK1/2 phosphorylation at a later time point (e.g., 24 h post CORT stimulation) (Fig 5D, 5E and 5F).

### CORT reduces PI3K/AKT phosphorylation in primary hippocampal neuron cultures

**Fig 6:**
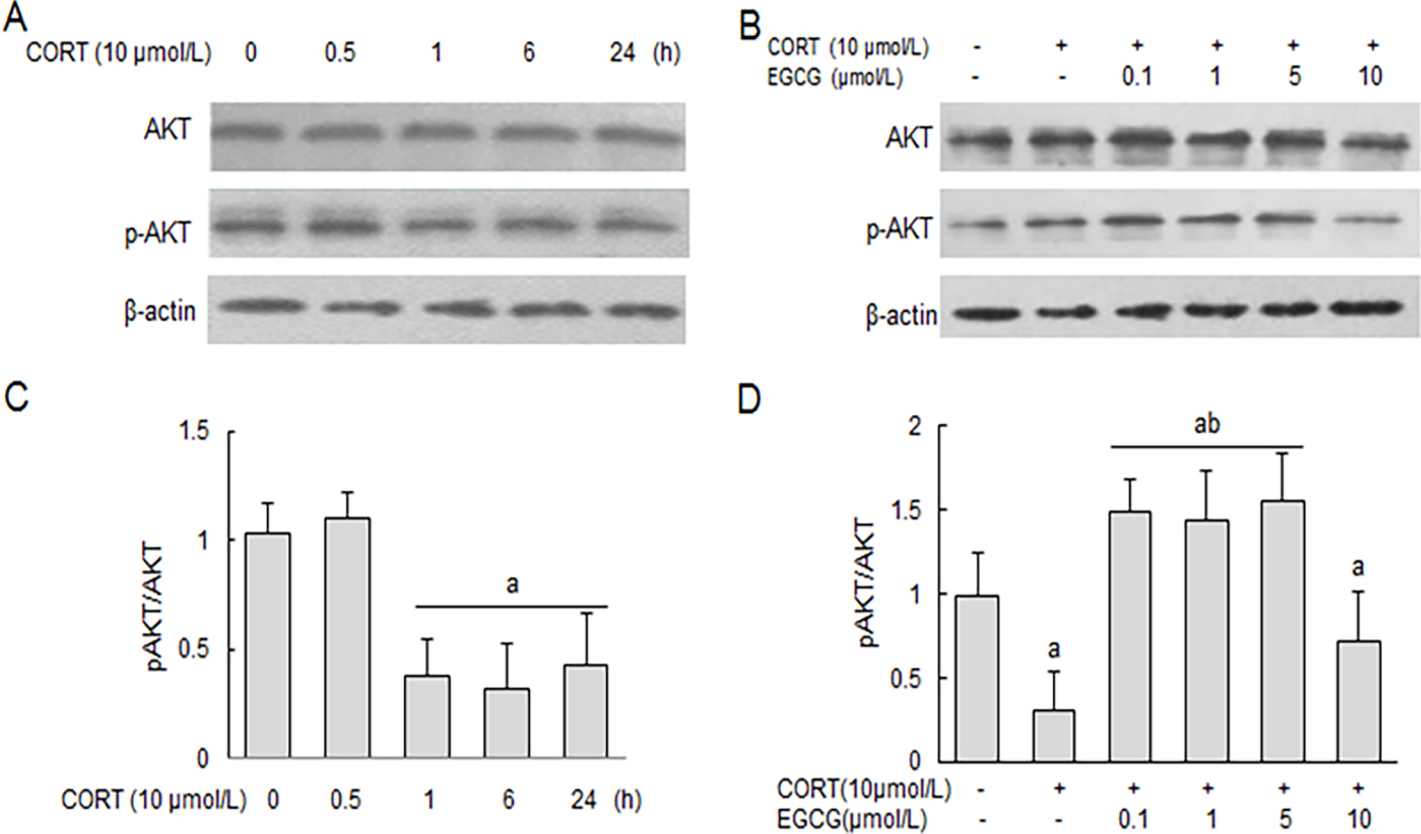
The effect of EGCG on CORT-induced decrease of AKT phosphorylation in primary hippocampal neuron cultures. Primary hippocampal neuron cultures were stimulated with CORT for 0, 0.5, 1, 6 and 24 h, respectively in the absence of EGCG (A), or in the presence of a pre-treatment with EGCG at indicated concentration for 2 h before CORT stimulation for 24 h (B). The cellular levels of total and phosphorylated AKT were determined by western blot analysis with reference to a house-keeping protein (β-actin) as loading control. AKT and The relative protein levels were expressed as the relative band density of the corresponding protein (C, D). ^a^ *P*<0.05 *vs* normal control group: ^b^ *P*<0.05 *vs* CORT treatment group.

When the primary hippocampal neurons were treated with corticosterone, the level of phosphorylated PI3K/AKT was significantly decreased (Fig 6A and 6C). However, pre-treatment with EGCG significantly prevented the CORT-induced decrease of PI3K/AKT phosphorylation between 0.1 and 5 μmol/L concentration while had no effect at 10 μmol/L level (Fig 6B and 6D).

### The expression of ERK1/2 and AKT mRNA in primary hippocampal neuron cultures

**Fig 7:**
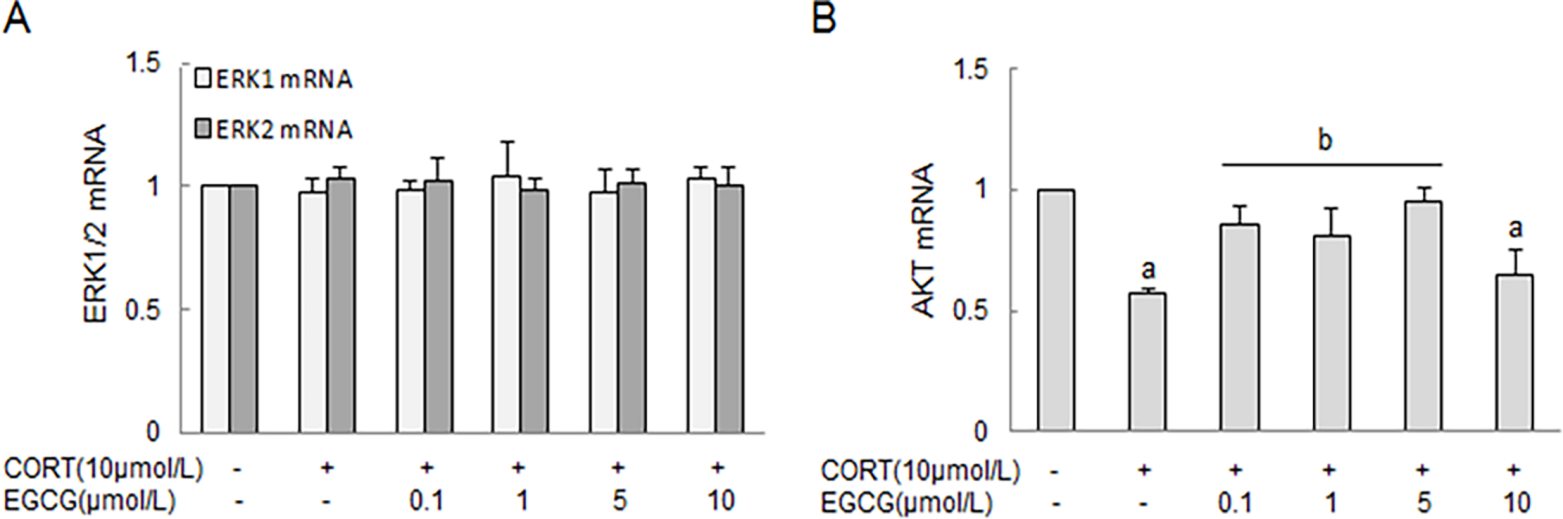
The effect of CORT and EGCG on ERK1/2 and AKT mRNA in primary hippocampal neurons. Primary hippocampal neuron cultures were pre-treated with EGCG for 2 h at indicated concentrations before stimulation with CORT for 24 h. The total RNA was isolated to measure the expression of ERK1/2 and AKT mRNA by RT-PCR, and expressed as mean ± SD of GAPDH mRNA levels. A) The expression of ERK1/2 mRNA; B) The expression of AKT mRNA. ^a^ *P*<0.05 *vs* normal control group: ^b^ *P*<0.05 *vs* CORT treatment group.

The expression of ERK1/2 and AKT mRNA in primary hippocampal neuron cultures were measured by RT-PCR. As shown in Fig 7, corticosterone and/or EGCG treatment at various doses did not alter the expression of ERK1/2 mRNA in the primary hippocampal neurons (Fig 7A). In contrast, corticosterone stimulation resulted in a significant reduction of AKT mRNA expression, but was dose-dependently attenuated by EGCG at concentrations between 0.1 and 5 μmol/L (Fig 7B). These changes of AKT mRNA were consistent with the aforementioned alteration of AKT phosphorylation following similar stimulation with EGCG and CORT.

### The changes of ATP production and PGC-1α expressions in primary hippocampal neuron cultures

**Fig 8:**
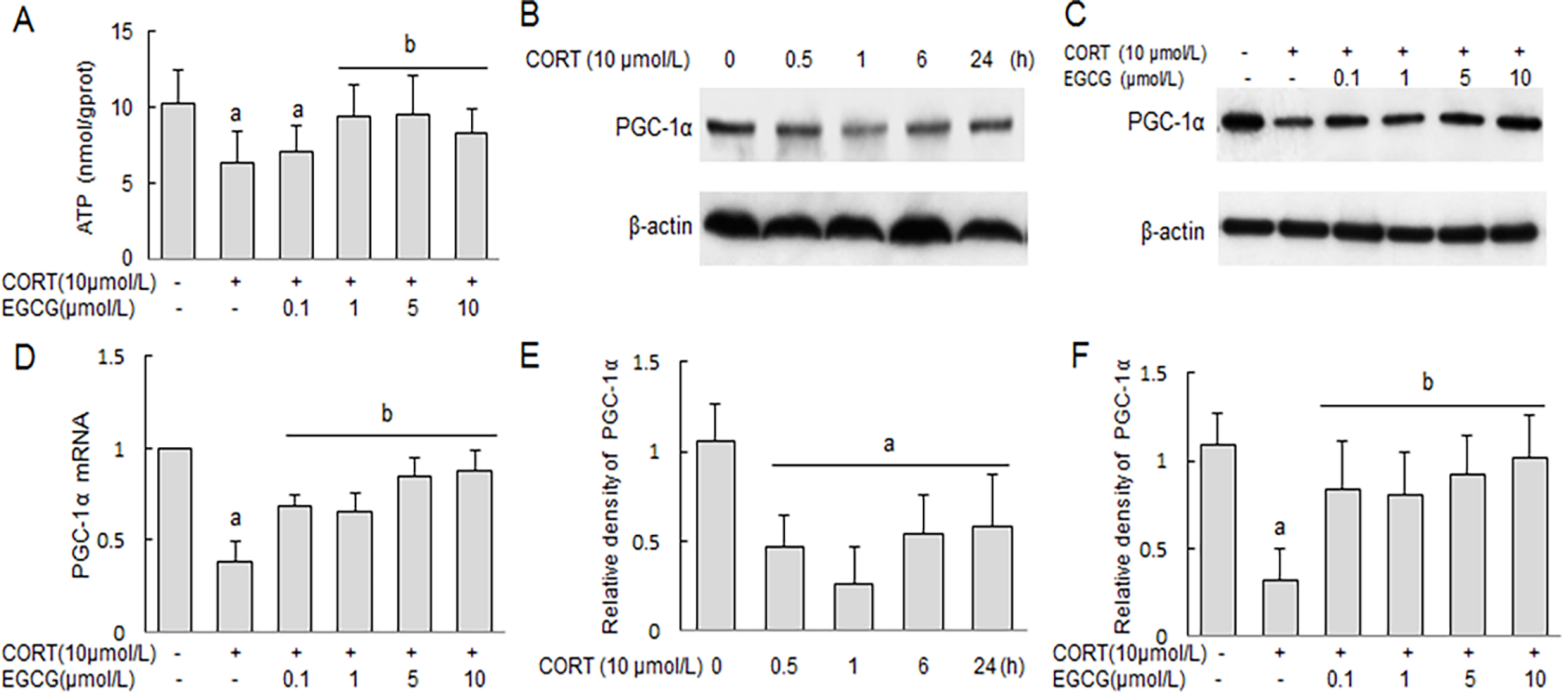
Effect of EGCG on CORT-induced changes of ATP and PGC-1α expressions in primary hippocampal neurons. Primary hippocampal neuron cultures were stimulated with CORT for indicated time period in the absence of presence of EGCG pretreatment (for 2 h) at indicated concentrations. The cultured hippocampal neurons were harvested and lysed to measure cellular ATP content, as well as PGC-1α protein and mRNA expression. The PGC-1α protein content was measured by western blot analysis, whereas the PGC-1α mRNA levels were determined by RT-PCR and expressed as mean ± SD of GAPDH mRNA level. A) The production of ATP; B, C) Examination of PGC-1α by western blot; D) The expression of PGC-1α mRNA; E, F) The relative expression of PGC-1α. ^a^ *P*<0.05 *vs* normal control group: ^b^ *P*<0.05 *vs* CORT treatment group.

Compared with normal control group, corticosterone stimulation resulted in a reduction of ATP production in primary hippocampal neurons (Fig 8A), which was dose-dependently attenuated by EGCG pretreatment (Fig 8A). Western blot analysis revealed a time-dependent decrease in the expression of PGC-1α in the primary hippocampal neurons following stimulation with corticosterone (Fig 8B and 8E), which was similarly attenuated by EGCG pre-treatment in a dose-dependent fashion (Fig 8C and 8F). Consistently, EGCG pretreatment similarly prevented CORT-induced down-regulation of PGC-1α mRNA in primary hippocampal neurons (Fig 8D).

## Discussion

In recent years, the ever-increasing psychological stress intensity and duration have brought about multiple stress-induced neural injuries which can dampen the risk of many neuropsychiatric disorders [27, 28]. Currently there are no effective strategies to prevent and treat such stress-induced neural injuries. Being endowed with various profitable properties, EGCG has been shown to possess powerful neuro-protective and neuro-rescue activities, which meets the demand for effective treatment for central neural dysfunctions. Before the potential therapeutic usage of EGCG clinically, more efforts should be made to elucidate the intricate mechanisms underlying the EGCG-mediated neuroprotection. In the present study, we provided evidence to support the notion that EGCG could protect against corticosterone-induced neural injuries in primary hippocampal neurons via restoring ERK1/2 and PI3K/AKT signaling pathways, as well as recovering the PGC-1α-mediated energy metabolism.

It is well-known that endogenic glucocorticoids (GCs) occupy an important role in the pathological impairments induced by stress and other insults [29, 30]. When exposed to psychogenic (e.g., fear or anxiety) or physical (e.g., cold, pathogen invasion or cellular lesion) stressors, the hypothalamic-pituitary-adrenal (HPA) axis is activated, resulting in the increased release of GCs to mount a critical physiological response to stress [31–33]. While GCs primarily act to maintain homeostasis by inducing physiological and behavioral adaptation, prolonged exposure to stress and elevated GCs levels may result in neuro- and psychopathology. Substantial experimental evidence has suggested that excessively elevated GCs levels and prolonged exposure to stressful conditions increase the susceptibility to develop behavioral impairments [4,5,34], metabolic, neuropsychiatric, and neurodegenerative disorders [35–37]. Furthermore, it also induces structural remodeling of neurons with synaptic loss as well as alterations in glial functions, which are frequently maladaptive [38]. As the prominent part of limbic system in the central nervous system, hippocampus plays a critical role in mediating behavioral, functional and neuroendocrine responses to stress [39]. Carrying abundant receptors for stress hormones, the hippocampus is also the main cerebral domain to mediate stress response, as well as the primary target affected by the release of stress hormones [40]. Therefore, hippocampus is highly sensitive to stress and vulnerable to the subsequent release of glucocorticoids. In the present study, we used corticosterone to develop a cell model of stress in the primary cultured hippocampal neurons, and confirmed that corticosterone induced obvious neural injuries in a dose-dependent manner in rat hippocampal neurons.

Meanwhile, substantial evidence has supported an association between EGCG’s capacity in modulating various signaling pathways and its substantial neuro-protective/neuro-restorative effects. For instance, we recently reported that the PKCα and ERK1/2 signaling pathways are involved in EGCG’s protection against stress-induced neural injuries in vivo [4]. In the present study, we sought to further evaluate EGCG’s impact on these signaling pathways using an *in vitro* cellular model of neuronal injury induced by excessive corticosterone exposure. Our results demonstrated that EGCG dose-dependently attenuated the corticosterone-induced neuron injuries, but the EGCG-mediated neuroprotective effects were impaired by the pharmacological inhibition of the ERK1/2 and PI3K/AKT signaling pathways. Mechanistically, EGCG pre-treatment significantly attenuated the corticosterone-mediated inhibition of ERK1/2 and PI3K/AKT phosphorylation, which are critical merging points for many signaling cascades involved in the regulation of cell survival, cell growth and proliferation under physiological and pathophysiologic conditions [41, 42]. EGCG has been shown to modulate ERK and PI3K/AKT signaling systems in different tissues and cell lines [1, 4, 43–45]. In the nervous system, a low level of ERK activation is needed to promote neuronal growth, thereby facilitating neuronal plasticity and survival [46]. However, the excessive or extremely prolonged activation of the ERK pathway can be deleterious, and contributes to the pathogenesis of various neurodegenerative disorders such as Parkinson`s and Alzheimer`s diseases [47–49]. The present study also showed that pretreatment with EGCG for 1 h could prevent the CORT-induced early inhibition of ERK1/2 in hippocampal neurons. While EGCG treatment could not affect CORT-induced ERK1/2 activation at a later time point (e.g., 24 h post CORT stimulation), which may contribute to the subsequent neuron injuries. In addition, our results showed that EGCG intervention significantly prevented corticosterone-induced decrease of AKT activation in hippocampal neurons. In response to a variety of extracellular factors (such as neurotropic factors, cytokines, hormones, neurotransmitters), PI3K is activated and further induces the phosphorylation of the serine–threonine kinase AKT, resulting in neuron proliferation, survival and differentiation [50, 51]. Multiple studies have demonstrated that EGCG could activate the PI3K/AKT signaling to improve learning and memory retention, inhibit sevoflurane-induced neurodegeneration [52], modulate neurogenesis and stroke recovery [53, 54], and promote cell growth and neuron differentiation [55]. Conceptually, the ERK1/2 and PI3K/AKT represent two independent parallel signaling pathways, which may cross-talk to regulate each other positively or negatively [56–59]. In differentiated human skeletal muscle cells, extracellular ATP could activate ERK1/2 phosphorylation which was strictly dependent on PI3K activity [60]. During brain ischemia/reperfusion, negative crosstalk exists between MAPK/ERK1/2 and PI3K/AKT pathways, which may be pharmacologically modulated for reducing neuronal injury [58]. Furthermore, once activated, ERK, AKT and other related kinases often act on common substrates, sometimes in concert to improve cell metabolism, proliferation, survival and motility [61, 62]. Collectively, the evidence strongly supports the notion that the activation of ERK1/2 and PI3K/AKT signaling pathways underlies the EGCG-mediated neuroprotective effects. In the future, more work is required to fully understand the specific interaction between MAPK/ERK and PI3K/AKT pathways, including the extent of their crosstalk and its significance in EGCG-mediated therapeutic potential in central neural injuries.

As a higher energy demanding system, the cerebrum is particularly vulnerable to dysfunctional energy metabolism, which impairs the nervous system development and contributes to the pathogenesis of various neurodevelopmental disorders [63–66]. We have recently demonstrated that the ATP production and the expression of a key energy modulator, PGC-1α, were significantly declined in the hippocampus and neocortex of stress animals [4]. In the present study, we further showed that ATP content and PGC-1α expression were both decreased in the corticosterone-stimulated hippocampal neurons, which was similarly attenuated by EGCG pretreatment. In agreement with the essential role of PGC-1α in the regulation of mitochondrial oxidative metabolism, respiration and mitochondrial biogenesis [67,68], our findings suggested that EGCG’s neuroprotection may be partly associated with its enhancement of PGC-1α expressions as well as the resultant ATP production in hippocampal neurons. In other neuronal cells (e.g., PC12), EGCG could suppress the 1-methyl-4-phenyl-pyridine (MPP)-induced oxidative stress by upregulating PGC-1α mRNA levels [69]. In hippocampal progenitor cells from a Down syndrome mouse model and cells derived from patients with Down’s syndrome, EGCG could also sustain and enhance mitochondrial functions by up-regulating PGC-1α/SIRT1/AMPK axis as well as increasing SIRT1-dependent PGC-1α deacetylation [64, 66]. In addition, the most recent studies suggested that PGC-1α could interact or collaborate with critical cellular signaling pathways, such as NF-κB [70], ERK1/2 [71], PI3K/AKT [72, 73], cAMP response element binding protein (CREB) [74] and AMPK [75] pathways, and collectively modulate mitochondrial functions in physiological and pathological conditions. So the modulation of intracellular pathways by EGCG, which ultimately restore mitochondrial functions, could explain, at least partly, the effects of such natural active substances in ameliorating the stress- or corticosterone- induced neural injuries.

In summary, the present report suggests that EGCG exerts neuro-protection against the corticosterone-induced neuron injuries via restoration of ERK1/2 and PI3K/AKT signaling pathways, and promotion of PGC-1α expressions as well as ATP production in the primary hippocampal neurons. This further verified the intricate molecular mechanisms underlying EGCG-mediated neuro-protection against stress/stress hormone-induced neural injuries. This research paves the way for using natural active substances as a potential therapeutic tool in preventing or managing stress-related and other diverse neural dysfunctions.

## Abbreviations

AKT: protein kinase B
AMPK: 5′ AMP-activated protein kinase
ATP: adenosine triphosphate
CORT: corticosterone
CREB: cAMP response element binding protein
EGCG: epigallocatechin-3-gallate
ERK1/2: extracellular signal-regulated kinase 1/2
GC: glucocorticoids
GTPs: green tea polyphenols
pAKT: phosphorylated protein kinase B
pERK1/2: phosphorylated extracellular signal-regulated kinase 1/2
PGC-1α: peroxisome proliferators-activated receptor-γ coactivator-1α
PI3K: phosphatidylinositol-3 kinase
